# Supplementary material for: High Glucosinolate Content in Rocket Leaves (Diplotaxis tenuifolia and Eruca sativa) after Multiple Harvests Is Associated with Increased Bitterness, Pungency, and Reduced Consumer Liking
Source: Foods. 2020 Dec 3;9(12):1799. doi: 10.3390/foods9121799 (PMC7761679; doi:10.3390/foods9121799)
Supplement: Supplementary file 1 [file foods-09-01799-s001.zip › Supplementary Table S1.docx]

| **Table S1.** Consumer demographics and characteristics for each of the rocket study panel months | | | | | | | |
| --- | --- | --- | --- | --- | --- | --- | --- |
| **Attribute** | **Jan** | **Mar** | **Apr/May** | **Jul** | **Sept** | **Nov** | **Average** |
| Total number of volunteers | 101 | 55 | 90 | 100 | 89 | 86 | ***87*** |
| *Age* |  |  |  |  |  |  |  |
| Mean | 35 | 35 | 33 | 36 | 35 | 33 | ***35*** |
| Median | 32 | 31 | 30 | 33 | 31 | 32 | ***32*** |
| Minimum | 21 | 19 | 21 | 20 | 19 | 18 | ***20*** |
| Maximum | 69 | 69 | 69 | 70 | 68 | 63 | ***68*** |
| *Gender* |  |  |  |  |  |  |  |
| Female (%) | 72.7 | 83.6 | 58.9 | 64 | 74.2 | 70.6 | ***70.7*** |
| Male (%) | 27.3 | 16.4 | 41.1 | 36 | 25.8 | 29.4 | ***29.3*** |
| *Work status* |  |  |  |  |  |  |  |
| Employed (%) | 61.6 | 50.9 | 34.4 | 58 | 46.1 | 54.1 | ***50.9*** |
| Food/nutrition students (%) | 20.2 | 25.5 | 44.4 | 31 | 31.5 | 10.6 | ***27.2*** |
| Other (%) | 1 | 0 | 3.3 | 1 | 2.2 | 0 | ***1.3*** |
| Student (%) | 31.3 | 49.1 | 62.2 | 41 | 51.7 | 45.9 | ***46.9*** |
| Unemployed (%) | 1 | 0 | 0 | 0 | 0 | 0 | ***0.2*** |
| Work in the food/nutrition/sensory sector(s) (%) | 11.1 | 18.2 | 21.1 | 20 | 14.6 | 15.3 | ***16.7*** |
| *Ethnicity/nationality* |  |  |  |  |  |  |  |
| African (%) | 4 | 5.5 | 6.7 | 7 | 4.5 | 1.2 | ***4.8*** |
| Caribbean (%) | 2 | 3.6 | 2.2 | 0 | 4.5 | 1.2 | ***2.3*** |
| Chinese (%) | 5.1 | 9.1 | 14.4 | 5 | 5.6 | 1.2 | ***6.7*** |
| Indian (%) | 2 | 1.8 | 4.4 | 2 | 3.4 | 2.4 | ***2.7*** |
| Mixed (%) | 3 | 1.8 | 0 | 0 | 0 | 1.2 | ***1*** |
| Other (%) | 31.3 | 32.7 | 35.6 | 17 | 22.5 | 27.1 | ***27.7*** |
| Not declared (%) | 2 | 1.8 | 2.2 | 6 | 5.6 | 2.4 | ***3.3*** |
| White (%) | 50.5 | 43.6 | 25.6 | 60 | 49.4 | 60 | ***48.2*** |
| *Rocket consumption frequency if available* |  |  |  |  |  |  |  |
| Never (%) | 6.1 | 18.2 | 4.4 | 2 | 1.1 | 0 | ***5.3*** |
| Rarely (%) | 12.1 | 10.9 | 23.3 | 9 | 9 | 11.8 | ***12.7*** |
| Sometimes (%) | 45.5 | 43.6 | 46.7 | 47 | 41.6 | 37.6 | ***43.7*** |
| Usually (%) | 26.3 | 18.2 | 22.2 | 30 | 40.4 | 37.6 | ***29.1*** |
| Always (%) | 10.1 | 9.1 | 3.3 | 12 | 7.9 | 12.9 | ***9.2*** |
