# Supplementary material for: High Glucosinolate Content in Rocket Leaves (Diplotaxis tenuifolia and Eruca sativa) after Multiple Harvests Is Associated with Increased Bitterness, Pungency, and Reduced Consumer Liking
Source: Foods. 2020 Dec 3;9(12):1799. doi: 10.3390/foods9121799 (PMC7761679; doi:10.3390/foods9121799)
Supplement: Supplementary file 1 [file foods-09-01799-s001.zip › Supplementary Table S2.docx]

| **Table S2.** Taste liking of rocket cultivars for the clusters of consumers obtained from agglomerative hierarchical clustering. | | | | | | | |
| --- | --- | --- | --- | --- | --- | --- | --- |
| **Month** | **Cluster** | **Cultivar** | | | | | **Average** |
|  |  | Tricia ◆🟁 | Yeti ◆🟅 | Wildfire ◼🟅 | Fast Grow ❖★ | Yeti ◆★ |  |
| **January** | 1 (n = 59) | 6.9 | 7.0 | 7.2 | 6.5 | 7.1 | **7.1** |
|  | 2 (n = 32) | 5.1 | 6.0 | 4.2 | 5.9 | 5.9 | **5.4** |
|  | 3 (n = 10) | 6.0 | 3.1 | 7.3 | 7.3 | 5.9 | **7.4** |
|  | All | 6.3 ns | 6.6 ns | 6.3 ns | 6.4 ns | 6.3 ns | **6.4 C** |
| **March** |  | Yeti ❖🟋 | Wildfire ◼🟅 | Fast Grow ◼- | Fast Grow ❖🟋 | Voyager ❖★ | **Average** |
|  | 1 (n = 17) | 3.2 | 6.1 | 7.7 | 5.2 | 5.7 | **5.6** |
|  | 2 (n = 8) | 3.0 | 2.9 | 6.4 | 3.1 | 6.5 | **4.4** |
|  | 3 (n = 21) | 6.5 | 5.8 | 6.4 | 6.8 | 6.0 | **6.3** |
|  | 4 (n = 9) | 4.9 | 3.2 | 4.8 | 4.0 | 1.7 | **3.7** |
|  | All | 4.7 a | 5.0 a | 6.5 b | 5.3 a | 5.3 a | **5.4 A** |
| **April/May** |  | Venere ◆🟅 | Wildfire ❖🟋 | Standard ❖🟅 | Giove ❖- | Quaggio ◆🟅 | **Average** |
|  | 1 (n = 31) | 4.8 | 6.1 | 5.1 | 6.4 | 5.5 | **5.6** |
|  | 2 (n = 24) | 5.7 | 2.8 | 4.9 | 3.3 | 5.4 | **4.4** |
|  | 3 (n = 35) | 7.3 | 5.6 | 7.1 | 5.5 | 7.5 | **6.6** |
|  | All | 6.0 bc | 5.0 a | 5.3 abc | 5.2 ab | 6.3 c | **5.6 A** |
| **July** |  | Giove ◼🟁 | Giove ◼🟁 | Giove ◼🟁 | Napoli ◼🟁 | Voyager ◼🟅 | **Average** |
|  | 1 (n = 58) | 6.2 | 6.5 | 7.0 | 6.6 | 7.0 | **6.7** |
|  | 2 (n = 23) | 6.0 | 6.3 | 3.0 | 6.3 | 6.8 | **5.7** |
|  | 3 (n = 19) | 4.9 | 5.5 | 4.3 | 4.9 | 2.7 | **4.5** |
|  | All | 5.9 ns | 6.2 ns | 5.5 ns | 6.2 ns | 6.2 ns | **6.0 B** |
| **September** |  | Voyager ◼🟅 | Shamrock ◼🟅 | Extrema ◆🟅 | Extrema ◆🟅 | Giove ◼🟁 | **Average** |
|  | 1 (n = 51) | 6.7 | 7.1 | 6.8 | 7.3 | 6.4 | **6.9** |
|  | 2 (n = 15) | 4.7 | 4.6 | 4.2 | 6.6 | 3.9 | **4.8** |
|  | 3 (n =23) | 5.7 | 6.7 | 5.8 | 3.3 | 6.6 | **5.6** |
|  | All | 6.1 ns | 6.6 ns | 6.1 ns | 6.1 ns | 6.0 ns | **6.2 BC** |
| **November** |  | Yeti ◼🟅 | Yeti ◼🟅 | Selezione Enza ◆🟅 | Tokita ◼🟁 | Tokita ◼🟁 | **Average** |
|  | 1 (n = 38) | 7.3 | 7.0 | 7.4 | 6.5 | 7.4 | **7.1** |
|  | 2 (n = 29) | 5.6 | 5.6 | 6.0 | 4.6 | 4.0 | **5.2** |
|  | 3 (n =19) | 6.8 | 6.3 | 3.2 | 6.6 | 7.4 | **6.1** |
|  | All | 6.6 ns | 6.3 ns | 6.0 ns | 5.9 ns | 6.3 ns | **6.2 BC** |
| Letters indicate ANOVA pairwise comparison significances (Tukey’s HSD). Lower case letters refer to individual cultivar scores across each consumer panel month. Upper case letters refer to monthly average scores. Where letters are different a significant difference was observed (*P*<0.0001).  ◼ = 1^st^ cut; ◆ = second cut; ❖ = 2nd+ cut; 🟁 = <30 day crop cycle; 🟅 = 31-60 day crop cycle; ★ = 61-90 day crop cycle; 🟋 = >91 day crop cycle; ns = no significant difference.  N.B. Hyphens (-) indicate data was not supplied from the grower. | | | | | | | |
