# Supplementary figures and images for: High Glucosinolate Content in Rocket Leaves (Diplotaxis tenuifolia and Eruca sativa) after Multiple Harvests Is Associated with Increased Bitterness, Pungency, and Reduced Consumer Liking
Source: Foods. 2020 Dec 3;9(12):1799. doi: 10.3390/foods9121799 (PMC7761679; doi:10.3390/foods9121799)

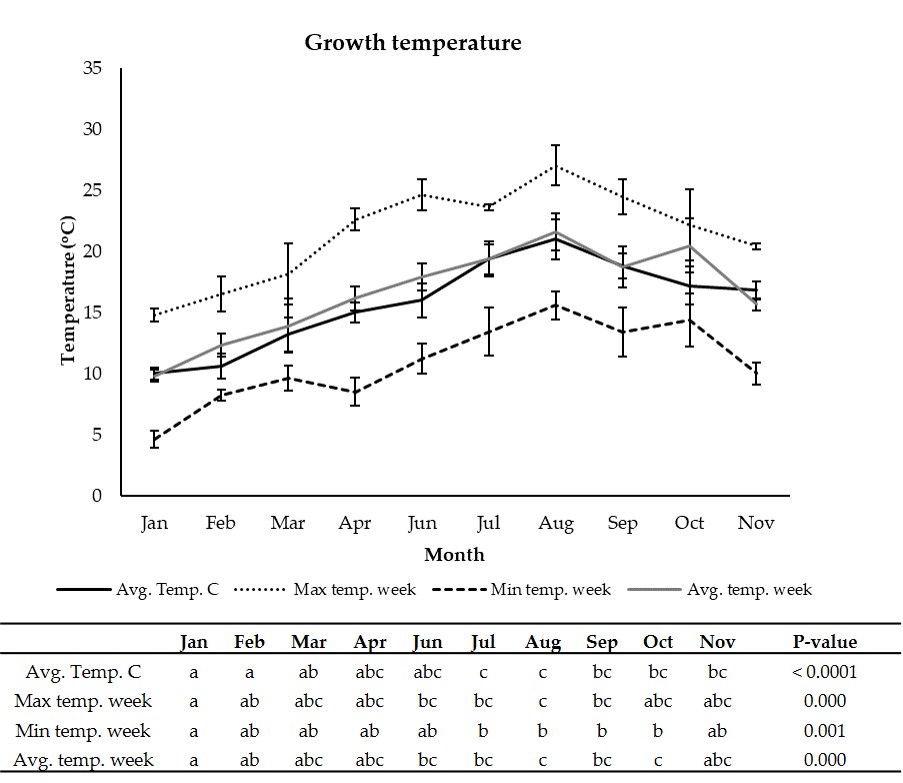

Supplement: Supplementary file 1 [file foods-09-01799-s001.zip › Supplementary Figure S1.jpg]

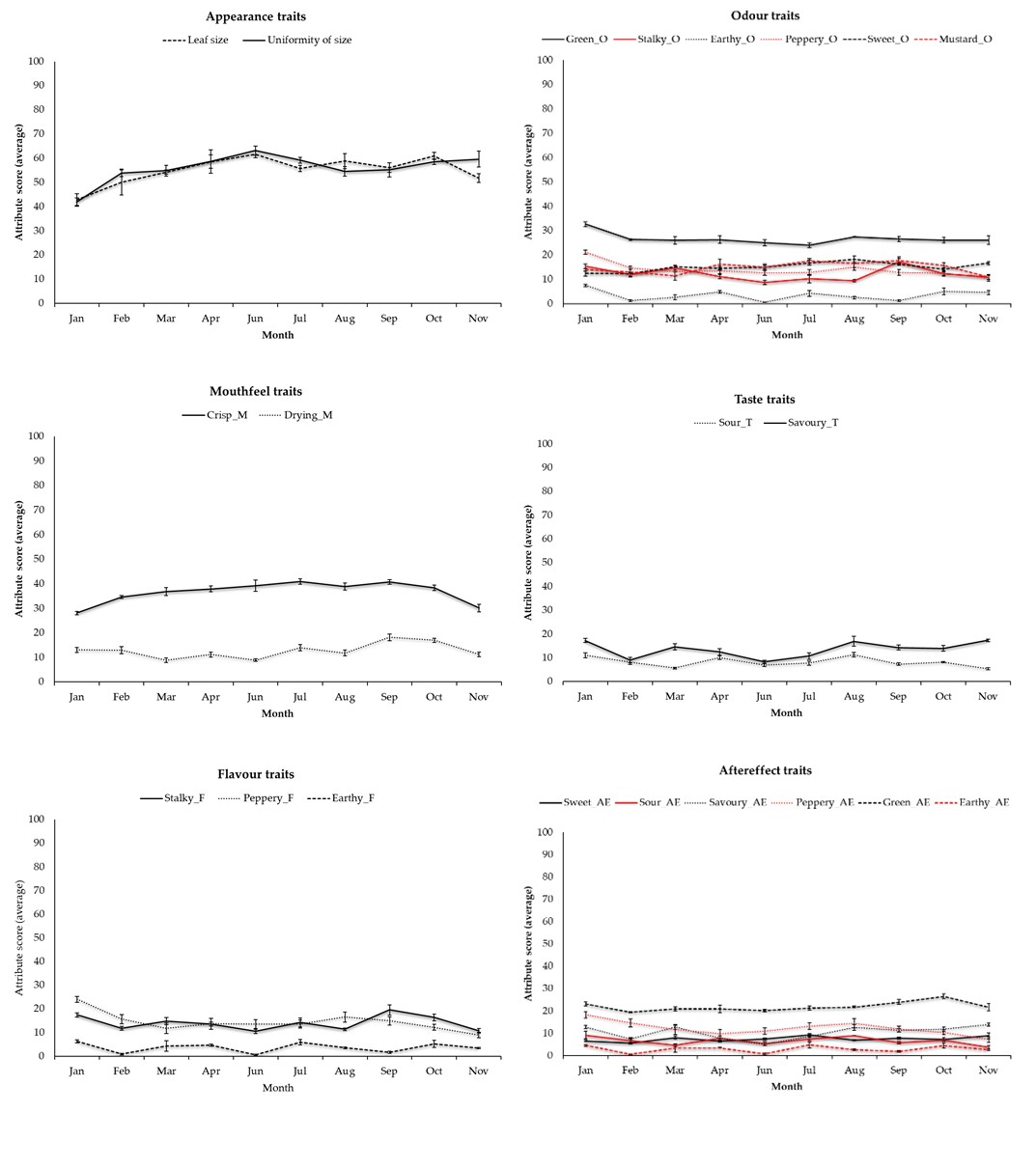

Supplement: Supplementary file 1 [file foods-09-01799-s001.zip › Supplementary Figure S2.jpg]
